# A SIMPLE METHOD OF DNA EXTRACTION OF *MYCOBACTERIUM TUBERCULOSIS* FROM SPUTUM CULTURES FOR SEQUENCING ANALYSIS

**DOI:** 10.21010/ajidv15i2S:2

**Published:** 2021-09-01

**Authors:** Maharani Pertiwi Koentjoro, Adyan Donastin, Endry Nugroho Prasetyo

**Affiliations:** 1 Medical Laboratory Technology, Faculty of Health, Universitas Nahdlatul Ulama Surabaya, Jl. Jemursari No. 57, Surabaya, Indonesia; 2 Faculty of Medicine, Universitas Nahdlatul Ulama Surabaya, Jl. Jemursari No. 57, Surabaya, Indonesia; 3 Departement of Biology, Faculty of Science and Data Analytics, Institut Teknologi Sepuluh Nopember, Gedung H, Kampus ITS Sukolilo, Surabaya, Indonesia

**Keywords:** DNA extraction, *Mycobacterium tuberculosis*, sequencing, sputum

## Abstract

**Background:**

Concern has been raised about DNA extraction from *Mycobacterium tuberculosis* due to its complex procedure. This study demonstrates a simple and fast DNA extraction method of mycobacterial genome to subsequent molecular investigation, such as Polymerization Chain Reaction (PCR) amplification, with species-specific primers and sequencing.

**Materials and Methods:**

Total DNA was isolated from *M. tuberculosis* cultured by using boil method. DNA was evaluated via measures of DNA quantity and quality (absorbance at 230, 260 and 280 nm), DNA integrity (electrophoresis). Molecular tests were tested namely PCR and sequencing.

**Conclusions:**

The quality of DNA obtained is acceptable for PCR and sequencing analysis. These findings demonstrate that the method used is inexpensive and suitable for minimum infrastructure facilities.

## Introduction

Tuberculosis (TB) is an infectious disease caused by *Mycobacterium tuberculosis* (Minggiano *et al.*, 2020). This bacterial infection is one of the top 10 causes of death worldwide. Furthermore, molecular analysis is a fast and sensitive diagnostic method for the detection and identification of these infections (Eddabra and Benhassou, 2018). High quality, pure DNA extraction protocol is a prerequisite for successful molecular investigations (Raschenberger et al., 2016). The DNA extraction process for *M. tuberculosis* is often time-consuming, and requires specialized material or facilities (Almedida *et al.*, 2013). Therefore, faster and simpler methods for DNA isolation help to complete the process in a considerable amount of time, using only equipment needed to perform molecular investigation (Bjorn-Mortensen *et al.*, 2015). This study proposed a simple alternative protocol for performing DNA extraction of *M. tuberculosis* directly from Löwenstein-Jensen (LJ) medium, which consisted of cultures from patients diagnosed with full-blown TB. The effectiveness of the extraction method was assessed using Polymerization Chain Reaction (PCR) and sequencing assays.

## Materials and Methods

### Clinical data

The sputum samples in this study were collected from patients undergoing treatment in the Hospital of Islam Jemursari (RSI), Surabaya-Indonesia, in the year 2020 (Table 1).

**Table 1:** Clinical data from patients included the positive control.

No samples	Symptoms	Positive TB	Origin
1	Cough to clarify	Yes	Surabaya, Indonesia

2	Cough to clarify	Yes	Surabaya, Indonesia

The study was admitted by Institutional Ethics Committee. Informed patient consent was obtained and approved.

### Growth of *Mycobacterium tuberculosis*

A total of 100 µL sputum samples were cultured in 5 mL of Löwenstein-Jensen culture medium and incubated at 37°C. H37Rv strain of *M. tuberculosis* were used as positive control (Cui *et al.*, 2012).

### DNA extraction protocol and quantitative assessment

For each sample, a total of 0.5 mL UltraPure™ Distilled Water (Invitrogen) was added to 1.5 mL tube. A colony of *M. tuberculosis* cultures was transferred to the tube and mixed via a vortex. The suspension was placed in a boiling water bath (100°C) for 3 minutes to destroy the cell membrane. Then, suspension centrifuged at 15.000 rpm for 5 min in 4°C. A 300 µL supernatant was transferred to a sterile tube and processed for DNA analysis by spectrophotometry for evaluation of nucleic acid purity (ideal ratios: A_260_/A_280_ ≥ 1.8 and A_260_/A_230_ = 2. The 1% agarose gel electrophoresis was used to evaluate DNA integrity.

### Polymerization Chain Reaction (PCR) and electrophoresis

PCR was performed in a final volume of 50 µL containing 25 µL of GoTaq® Master Mixes (Promega); The ripA (Rpf-interacting protein A) gene in *M. tuberculosis* was amplified by ripA forward primers (5’–A TTCCGTCGGCTTTGAGTGT–3’) and ripA reverse primers (5’–CGGCGGATCACGTA TTCAGA–3’) (Koentjoro *et al.*, 2019). RipA gene is responsible for the cell division and cellular integrity of *M. tuberculosis* by cleave of the peptidoglycan layer connecting daughter cell (Maitra *et al.*, 2019). This gene expressed the endopeptidase ripA. In the absence of these genes, *M. tuberculosis* is unable to assemble the complex cell envelope. Therefore, ripA gene became the target of several anti-mycobacterial drugs. Amplification was carried out for 35 cycles, each consisting of initial denaturation at 92°C for 2 min, final denaturation at 92°C for 30 seconds, annealing at 92°C for 30 seconds, extension at 92°C for 1 minute, followed by a final extension at 92°C for 5 min. Additionally, PCR products were analysed by gel electrophoresis in 2% agarose gel. Target DNA fragments of 912 base pairs (bp) were viewed under UV light.

### Sequencing analysis

For validation, ripA genes from PCR reaction were sequenced by Macrogene Inc (Korea) using Sanger sequencing method. Data sequences correlated with the specified sequences on the NCBI BLAST web service (https://blast.ncbi.nlm.nih.gov/Blast.cgi).

## Results

**Figure 1:**
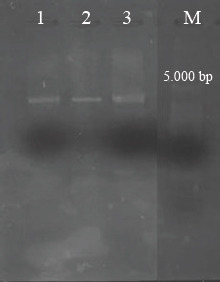
One percent (1%) Agarose gel electrophoresis of DNA aliquot. Lane 1 = *M. tuberculosis* H37Rv, Lane 2 = Sample 1, Lane 3 = Sample 2, M = Molecular marker 500 bp DNA

The DNA genome from M. tuberculosis were determined on 1 % agarose gel. Figure 1 shows the electrophoresis of DNA aliquots through heat extraction. Sharp bands and good resolution on agarose gel electrophoresis were observed, with no apparent signs of degradation. DNA quality and quantity are important parameters for a downstream molecular test.

The integrity of nucleic acids was assessed on an agarose gel and the purity calculated by the ratio of absorbance at 260 and 280 nm (A_260_/A_280_) respectively, with values ≥ 1.8 indicating highly pure samples (Sellin et al., 2014). Table 2 demonstrates A260/A280 and A260/A230.

**Table 2:** Average sample absorbance

No samples	A_260_/A_280_	A_260_/A_230_	Concentration (µg/µL)
*M. tuberculosis* H37Rv	1.94	2.10	276

Sample 1	1.63	1.89	250

Sample 2	1.73	1.99	230

**Figure 2:**
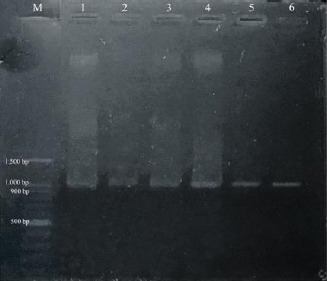
Two percent (2%) Agarose gel electrophoresis of ripA gene amplifications performed. Lane 1 = Molecular marker 100 bp DNA, Lane 1-2 = *M. tuberculosis* H37Rv, Lane 3-4= Sample 1, Lane 5-6 = Sample 2.

The aliquot was used as a DNA source for PCR, and the ripA gene was discovered to be successfully amplified (Figure 2, lanes 1-6). These findings indicate that the heat extraction method produces high-quality DNA from *M. tuberculosis* cultures samples for PCR.

**Figure 3:**
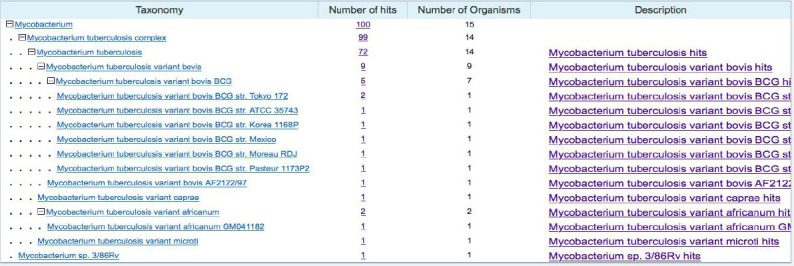
BLAST analysis of *M. tuberculosis* H37Rv ripA gene PCR product sequences.

Moreover, the DNA samples from this method were used to perform nucleotide acid sequencing. Figure 3 shows that sequence alignment was generated through Sanger sequencing. Therefore, the results obtained show that sequence data have good matches with those in NCBI. This shows that DNA aliquot from heat-extraction is good for sequencing analysis.

## Discussion

To provide an efficient and low-cost method for the extraction of DNA from *M. tuberculosis* quantitatively and qualitatively for molecular investigation, the DNA extraction procedure was modified for *M. tuberculosis*. This is necessary due to the fact that not every laboratory has complete facilities and DNA extraction kits. Therefore, this new procedure was performed to obtain DNA from *M. tuberculosis* via boil method. The effect of heat treatment destroyed *M. tuberculosis* (Cardona *et al.*, 2015). Valenta *et al*. (2009) reported that heat lyses the cell membrane of *Mycobacterium smegmatis* which results in the release of DNA. Spectrophotometer was used to analyze DNA quantity and concentrations ranging from 276 µg/µL. The time required to complete the entire DNA extraction procedure was only 30 minutes. This result is relatively quick and simple than extraction method using kit (Aldous *et al.*, 2005).

Several studies had discovered a comparison method of extracting *M. tuberculosis* DNA. Miyata et al., (2021) revealed that cetyltrimethylammonium bromide (CTAB) and thermolysis method was sufficient to extraction of *M. tuberculosis* DNA. These methods require 2 to 30 hours to obtained the DNA. Aldous *et al.*, (2005) reported that IDI lysis tubes showed the simple procedure, low cost, and time to require to complete the entire DNA extraction in less than 1 hours. Although very easy to do extraction using kit, it is expensive. Therefore, our method is promising to be applied in routine clinical practice.

This is a preliminary study with few samples. Furthermore, due to the costs of molecular tests in developing countries and the unavailability of diagnostic tools in every laboratory (Ssengooba et al., 2015), this simple method used to extract DNA of *M*. tuberculosis, becomes a viable alternative. This extraction procedure would be ideal for laboratories in Biosafety Level-3 (BSL-3) (Ssengooba et al., 2015), and laboratory staff will be safe at work with lesser exposure time with *M. tuberculosis*. Additionally, this result proposes a suitable method for other forms of molecular analysis.

## Conclusion

It is concluded that the DNA extraction protocol employed for *M. tuberculosis* from clinical sputum culture in the present study was useful and successful for performing molecular analysis such as PCR amplification and sequencing with species-specific primers. Additionally, the protocol is useful in laboratory settings with minimal infrastructure, less reagents and reduction in the risk of infection to the laboratory staff.

### Conflict of interests

**The authors declare that there is no conflict of interest associated with this study.**

**List of Abbreviations:**
ripA: Rpf-interacting protein A
TB: Tuberculosis
PCR: Polymerization Chain Reaction
